# Cognitive Correlates of Reading Fluency in Chinese School-Aged Children

**DOI:** 10.3389/fpsyg.2020.00903

**Published:** 2020-06-04

**Authors:** Jing Bai, Wenlong Li, Yang Yang, Jianhui Wu, Wei He, Min Xu

**Affiliations:** ^1^College of Psychology and Sociology, Shenzhen University, Shenzhen, China; ^2^Center for Brain Disorders and Cognitive Science, Shenzhen University, Shenzhen, China; ^3^Center for Language and Brain, Shenzhen Institute of Neuroscience, Shenzhen, China; ^4^School of Biomedical Engineering, Health Science Center, Shenzhen University, Shenzhen, China; ^5^CAS Key Laboratory of Behavioral Science, Center for Brain Science and Learning Difficulties, Institute of Psychology, Chinese Academy of Sciences, Beijing, China; ^6^Nanshan Educational Science Research Institute, Shenzhen, China

**Keywords:** reading fluency, Chinese, rapid automatized naming, writing fluency, phonological awareness, visual crowding

## Abstract

Previous studies have showed that reading fluency is strongly associated with cognitive skills, including rapid automatized naming, phonological awareness, orthographical awareness, and so on. However, these studies are largely based on alphabetic languages, and it remains unclear which cognitive factors contribute to the development of reading fluency in logographic Chinese, a language in which the graphic forms map onto morphemes (meaning) rather than phonemes. In Study 1, we tested 179 Chinese children aged 6 to 9 on a set of cognitive tasks as well as for word reading accuracy and sentence reading fluency. The results showed that rapid naming, writing fluency, and phonological awareness significantly predicted reading fluency in both beginning and intermediate readers. In addition, while the contribution of rapid naming and writing fluency increased with grades, the effect of phonological awareness decreased. In Study 2, we examined the role of visual crowding in reading fluency in a subgroup of 86 children and found that visual crowding accounted for the unique variance of individual differences in reading fluency. The findings reflect both universal and language-specific cognitive correlates of reading fluency and provide important implications for potentially effective treatment for individuals suffering from Chinese reading disabilities, particularly in terms of reading fluency.

## Introduction

Reading fluency, the ability to read rapidly and automatically, is considered a key to good reading comprehension (Nathan and Stanovich, 1991; Hudson et al., 2005; Katzir et al., 2006; Palmer, 2010). Differences in reading fluency distinguish good readers from poor readers. While good readers are able to recognize words efficiently and automatically and devote most of their cognitive resources to comprehending the text, poor readers tend to read in a labored, disconnected way, with most of their cognitive capacity focused on decoding at the word level (Perfetti, 1985; Hudson et al., 2005). Achieving reading fluency involves the efficient integration of information from different levels of language processing (including orthographic, phonological, semantic, and syntactic processes) with both great accuracy and speed (Norton and Wolf, 2012). In the past few decades, a large body of studies have explored which cognitive and linguistic factors account for individual differences in reading fluency. Evidence has accumulated to indicate that reading fluency is strongly associated with a complex system of cognitive skills, including rapid automatized naming (RAN) (Savage and Frederickson, 2005; Norton and Wolf, 2012; Georgiou et al., 2016; Strappini et al., 2017), phonological skills (Poulsen and Elbro, 2013; Kibby et al., 2014; Elhassan et al., 2017), orthographic skills (O’Brien et al., 2011; Ehri, 2013), and so on.

Rapid automatized naming involves naming a series of familiar items (such as letters, digits, colors, and objects) presented repeatedly in random order as quickly as possible. The important role of RAN in reading fluency may be due to the cognitive components that it shares with reading fluency, such as general processing speed, eye saccades, phonological process, and so on. In addition, the automaticity that supports RAN is also an important characteristic of fluent reading (Norton and Wolf, 2012). Savage and Frederickson (2005) tested 67 children and found that while phonological processing tasks predicted reading accuracy, rapid digit naming predicted both reading accuracy and rate, suggesting that RAN was a highly specific predictor of reading fluency (Savage and Frederickson, 2005). In another study, Georgiou et al., 2016 found that the contribution of rapid naming to reading fluency was greater than those of phonological awareness and orthographic awareness. Furthermore, the centrality of rapid naming in reading fluency has been found to be consistent across languages (Tan et al., 2005; Song et al., 2016; Landerl et al., 2019). For example, Landerl et al. (2019) found that rapid naming was a consistent predictor of reading fluency in English, French, German, Dutch, and Greek.

Phonological awareness (PA) refers to awareness of the phonological structure or sound structure of language (Wagner and Torgesen, 1987). It is evaluated with various tasks that require the subject to identify or manipulate phonological units (Sodoro et al., 2002; Hu, 2003; Kirby et al., 2008). Some researchers suggest that PA shares variance with RAN, but others argue that PA and RAN are independent processes that predict decoding accuracy and reading speed, respectively (Wolf and Bowers, 1999; Jones et al., 2009). Moreover, the relationship between PA and reading fluency is controversial. While there are studies demonstrating an important role of PA in reading fluency (Rakhlin et al., 2014), some other studies report that its contribution to reading fluency is small or restrained to the early stage of reading development (Elhassan et al., 2017). For example, Rakhlin et al. (2014) tested 96 Russian-speaking children (mean age = 13.73 years) on reading fluency, orthographic skills, PA, and RAN, and they revealed that PA accounted for more variance in reading fluency than orthographic awareness and RAN. Kibby et al. (2014) tested 182 English-speaking children aged 8–12 years and found that PA was the best predictor of reading fluency among variables including rapid letter naming, working memory, and attention. In contrast, Georgiou et al. (2008) tested 110 English-speaking Grade 1 children (mean age = 6.6 years) and 70 Greek-speaking Grade 1 children (mean age = 6.9 years), and the children were reassessed on word decoding and reading fluency when they were in Grade 2. They found that the impact of PA on reading fluency was smaller than on reading accuracy, and it was strong only for early reading acquisition in Grade 1. Differences in the roles of PA in reading fluency might be partly due to the different degrees of orthographic transparency of the languages involved in previous studies. For instance, Georgiou et al. (2008) suggested that PA was a stronger predictor of reading fluency in English than in Greek (relatively transparent orthography). In addition, the extent to which the PA measures captured the grain sizes that the readers relied upon during the reading fluency tasks might also impact the results.

Relative to RAN and PA, fewer studies have examined the role of orthographic awareness in reading fluency. Orthographic awareness refers to the ability to form, store, and access printed words and the general attributes of writing systems (such as order dependencies, structural redundancies, letter position frequencies, and so on), which are mainly obtained implicitly (O’Brien et al., 2011; Rakhlin et al., 2014; Elhassan et al., 2017). There are studies showing that orthographic awareness is closely correlated with the development of automatic word recognition that supports reading fluency. O’Brien et al. (2011) tested orthographic awareness, RAN, processing speed, reading, and spelling in 45 English speakers, who were in Grades 1 to 3. They found that orthographic awareness was related to reading speed for passages but not spelling performance (O’Brien et al., 2011). Papadopoulos et al. (2016) found that PA and orthographic awareness were significant predictors of reading fluency. In a recent study with a large sample size (*n* = 1491), Rakhlin et al. (2019) measured children’s reading fluency, reading accuracy, orthographic processing skill, phonological skills, RAN, and non-verbal intelligence. They found that orthographic skill was the strongest predictor of reading fluency in both good and poor readers (Rakhlin et al., 2019).

Furthermore, previous studies have demonstrated that the cognitive skills contributed differently to reading fluency in languages with different degrees of orthographic transparency (Georgiou et al., 2008; Vaessen et al., 2010; Pasquarella et al., 2015; Landerl et al., 2019). Transparent (or shallow) orthographies have one-to-one orthography–phonology correspondence, whereas opaque (or deep) orthographies have letters or letter strings that do not consistently map onto specific sound units. In a longitudinal study over a 2-year period, Landerl et al. (2019) measured students who were native speakers of one of five alphabetic orthographies with different degrees of orthographic consistency (French, English, German, Dutch, and Greek). They found that RAN predicted reading fluency in all orthographies, whereas the effect of PA on reading fluency depended on developmental stage and orthographic transparency. Specifically, children’s PA at the beginning of Grade 1 predicted reading fluency at the end of Grade 1 in French, which has deep orthography, but in German, the predictive pattern became apparent in Grade 2. In contrast, PA did not predict reading in Greek and Dutch, which were transparent orthographies. The findings were consistent with Ziegler et al. (2010) study, which showed that the contribution of PA to reading was greater in more opaque orthographies. In another study, Vaessen et al. (2010) investigated the relationship between cognitive skills and reading fluency in Grades 1–4 in three languages with different orthographic transparencies (Hungarian, Dutch, and Portuguese). They found that the contribution of PA to reading fluency was significant in all grades but decreased as the grades increased, and conversely, the contribution of RAN was increased with grade.

Moreover, the verbal efficiency model proposed by Perfetti (1977, 1985) has been considered as the basis for the theory of fluent reading (Meyer and Felton, 1999; Breznitz, 2006), which emphasizes the importance of lexical processes (such as phonological and orthographic processes) and working memory for enhancing reading effectiveness. It posits that fluent readers are efficient and can free working memory capacity to focus on the higher levels of reading. Working memory involves storing information temporarily and using the information in complex cognitive activities (Baddeley, 2003). Previous studies have found that verbal working memory plays an important role in reading comprehension and contributes unique variance to word reading and reading fluency (Shankweiler and Crain, 1986; McCallum et al., 2006; Georgiou et al., 2008). For example, Leong et al. (2008) study based on 518 Chinese children found that verbal working memory had strong effects on pseudoword reading and text comprehension.

In the present study, we investigated what cognitive skills contribute to the development of reading fluency in Chinese children. Written Chinese has an extremely opaque orthography, and it provides an important window for understanding the universal and writing system-specific cognitive processes that underlie the development of reading fluency (Perfetti et al., 2013). According to Perfetti’s verbal efficiency model, we focused on both lexical processes and cognitive capacities. We tested the roles of cognitive factors (including PA, RAN, orthographic awareness, verbal working memory, and executive control) in reading fluency. Moreover, based on the unique properties of Chinese characters, we examined whether the factors of writing fluency and visual crowding had unique contributions to Chinese reading fluency. The roles of writing fluency and visual crowding in Chinese reading fluency had seldom been tested in previous research, and therefore, our study went one step forward to give a more comprehensive picture of what cognitive factors contribute to the development of reading fluency in Chinese.

Chinese is a morpho-syllabic system, in which the graphic forms (characters) map onto morphemes (meaning) rather than phonemes (Siok et al., 2004). This means that there is no stroke or component in a character (e.g., 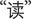,/du/, to read) that corresponds to a specific phoneme (e.g., /d/) in the syllable. Chinese characters are salient visual units, formed with intricate strokes within square configurations, which is in contrast to the linear structure of alphabetic words. Chinese characters can be categorized into simple characters and compound characters. Simple characters are formed with stroke patterns that cannot be decomposed (e.g., 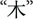,/mu4/, wood; 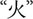,/huo3/, fire). The vast majority of characters are compound characters, which are formed with two or more identifiable components. About 85% of Chinese characters are phonetic compounds that contain a semantic radical and a phonetic radical, which, respectively, provide the semantic and phonological information of the characters. For example, in the character 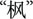 (/feng1/, maple), the semantic radical 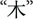
(/mu4/, wood) suggests that it is related to wood, and the phonetic radical 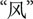
(/feng1/, wind) suggests its pronunciation/feng1/.

Previous studies on the development of reading fluency in Chinese mainly focused on cognitive factors including RAN, PA, orthographic awareness, and morphological awareness. For example, Liu et al. (2017) examined the role of cognitive factors such as PA, RAN, orthographic awareness, morphological awareness, and so on in reading development in 1776 Chinese children who were in Grades 1 to 6. The study used a word reading fluency test, in which the subjects read aloud a list of two-character words as fast as possible within one minute. The authors found that the effect of RAN on word reading fluency was strong and increased across age groups, whereas the effect of PA was significant only in the beginning readers (Liu et al., 2017). In another study, Xue et al. (2013) used a sentence-reading fluency task in which the children were asked to decide whether the statements in a series of sentences were true or not within 3 min. They found that character naming and RAN were the two most significant contributors to reading fluency among measurements including character naming, PA, RAN, orthographic awareness, morphological awareness, and verbal short-term memory.

Although some previous studies have emphasized the importance of writing ability in reading development (Guan et al., 2011; Tso et al., 2012; Tan et al., 2013), the role it plays in reading fluency has seldom been examined. One exception was the study by Tan et al. (2005), which investigated the relationship between cognitive skills (including RAN, PA, and writing fluency) and word reading fluency in 131 Chinese children. Reading fluency was measured as the number of characters read correctly within 2 min. They found that writing skills were strongly associated with word reading, while the contribution of PA to reading was minor and fragile (Tan et al., 2005). It was suggested that, through writing, children learned to deconstruct Chinese characters into strokes and stroke patterns and then regrouped these stroke patterns into a square unit, and, with practice, to establish long-term motor memory of Chinese characters. The current study extended Tan’s study by including the additional factors of orthographic processing, verbal working memory, executive control, and visual crowding.

In addition, due to the salient visual-orthographic property, it has been long proposed that reading of Chinese words is more prone to the influence of visual factors (Siok and Fletcher, 2001; Chung et al., 2008). As for reading fluency, one potentially critical visual factor that influences reading rate is visual crowding, which refers to impaired recognition of a target due to the presence of neighboring objects in the periphery. It occurs when the center-to-center distance of the two objects is smaller than the critical spacing, which is the distance between objects at which target recognition is restored (Bouma, 1970; Toet and Levi, 1992; Pelli, 2008). Previous research suggested visual crowding to be a critical condition that affects reading rates (Pelli et al., 2007). Crowding severely affected the peripheral vision of children and adult readers (Gori and Facoetti, 2015; Strappini et al., 2017) and slowed down reading processing (Cheung and Cheung, 2017). In addition, the crowding effect was particularly strong in dyslexic readers (Martelli et al., 2009). It was found that increasing the word and line spacing between English letters promoted reading fluency in dyslexic children (Zorzi et al., 2012) and improved reading accuracy in normal school-age children (Hakvoort et al., 2017). Previous research suggested that the size of the Chinese character visual span, i.e., the maximum number of characters that could be recognized without moving the eyes, was affected by the complexity of the characters and was mediated in large part by visual crowding (Wang et al., 2014). Therefore, we expected that the visual crowding effect would account for unique variance in Chinese reading fluency.

In Study 1, we used a cross-sectional design to examine the contributions of a set of cognitive skills to reading fluency in 179 first- to fourth-year primary school students. The cognitive factors included measures of RAN, PA and orthographic awareness, writing fluency, verbal working memory, and executive control. In Study 2, we further investigated whether the effect of visual crowding accounted for unique variance in Chinese reading fluency in a subgroup of children (*n* = 86) in Study 1.

## Study 1: the Effect of Cognitive Skills on Reading Fluency in Chinese Children

### Methods

#### Participants

One hundred and seventy-nine primary school children participated in the study. They were students of grades 1 through 4 at an elementary school in Shenzhen, China. In China, the focus of reading training in Grades 1 and 2 was recognition of words, whereas the focus transit to text reading in Grade 3, and therefore we divided the subjects into two groups: the beginning readers were 1st and 2nd graders (*n* = 91, *M*_age_ = 6.82, *SD* = 0.7), and the intermediate readers were 3rd and 4th graders (*n* = 88, *M*_age_ = 8.85, *SD* = 0.7) (Table 1). All the children were native speakers of Mandarin, which is the official dialect of Mainland China and the language of instruction in school. Of the subjects, 173 were right-handed, 3 were left-handed, and 3 were ambidextrous, as assessed by the handedness inventory (Oldfield, 1971).

**TABLE 1:** Demographic characteristics of the participants and descriptive statistics of the tests.

	**Beginner (Grades 1 and 2)**	**Intermediate (Grades 3 and 4)**	***t*-test**
Age, year	6.82 (0.7)	8.85 (0.7)	−19.66***
Gender (boy/girl)	54/37	54/34	0.275
Sentence reading fluency	19.07 (12.7)	41.6 (14.0)	−11.28***
Chinese word reading	62.05 (47.1)	163.4 (51.9)	−13.695***
RAN-D, sec	31.90 (7.9)	22.32 (5.4)	9.513***
RAN-O, sec	27.58 (5.7)	22.37 (4.5)	6.787***
RAN-C, sec	33.98 (11.3)	27.8 (6.6)	4.477***
PA	9.49 (5.4)	12.93 (4.6)	4.592***
Forward digit span	6.69 (1.0)	7.67 (1.0)	−6.368***
Backward digit span	3.19 (1.4)	4.01 (1.2)	−4.175***
Stroop, sec	20.1 (11.5)	21.27 (9.3)	–0.742
Chinese component search	23.32 (7.6)	31.15 (6.8)	−7.287***
Writing	23.24 (11.1)	49.68 (8.8)	−17.623***
Standardized non-verbal IQ	60.22 (28.9)	67.44 (23.7)	–1.831

Asterisk(s) indicates significance levels: *p < 0.05, **p < 0.01, and ***p < 0.001.

#### Procedure and Tasks

A battery of tests was administrated, including RAN, PA, orthographic awareness, writing fluency, verbal working memory, Stroop task, and non-verbal intelligence. The standardized Chinese version of Raven’s Standard Progressive Matrices was used as a non-verbal intelligence test. The reading performance was assessed with a word reading test and a sentence reading fluency test. All tests were conducted by trained experimenters. Written consent was obtained from the children’s parents before the tests. The non-verbal intelligence test, lasting about 40 min, was administered on a group basis, and the other tests were tested individually, taking about 40 min in total.

##### Rapid automatized naming (RAN)

The RAN tasks examined children’s ability to name letters, symbols, words, or objects in a quick and automatic manner. Previous research suggested that the contribution of RAN to reading development seemed to be dependent on the RAN tasks employed in the studies (e.g., Schatschneider et al., 2004; Georgiou et al., 2008). Hence, we conducted three subtests, digit naming (RAN-D), object naming (RAN-O), and color naming (RAN-C), to test whether different RAN tasks contributed differently to Chinese reading and in children of different reading levels. RAN-D involved the naming of digits (1–9) that were arranged in five rows and ten columns. RAN-O involved the naming of simple drawings of objects (watch, house, plane, glasses) that were arranged in four rows and seven columns. RAN-C involved the naming of colors (red, yellow, blue, and green) with color squares that were arranged in six rows and five columns. The subjects were required to name the stimuli in a left-to-right fashion as correctly and rapidly as possible. Time (in milliseconds) and accuracy of naming were recorded, but because the key variable of RAN was the time taken to name the items, only the time taken for naming was analyzed. There was a pre-test trial before each RAN subtest, in which the subjects were asked to name the stimulus items individually to ensure that they could accurately name the items. For each subtest, the subjects were given two separate trials, and the trial with the shorter total naming time was taken as the test score.

##### Phonological awareness (PA)

An oddity test was used to examine children’s phonological awareness of onsets and rimes. The children were given four practice items and twenty testing items, among which ten items tested onset awareness and ten tested rime awareness. We obtained a score for PA by adding the number of correct items for onset awareness and rime awareness. In each item, the children were required to carefully listen to four monosyllabic words. One of the four syllables was the odd one out by virtue of lacking an initial or final sound shared by the other three syllables. The subjects were asked to identify the odd one out. For example,/sha1/was the odd one out in the set/chu3/,/sha1/,/pu2/,/lu3/(the numbers indicate the tone of the syllables) in terms of the final sound. The score was the total number of items answered correctly. The maximum score for this task was 20.

##### Orthographic awareness

We used a component search task to test children’s awareness of the internal structure of Chinese characters. This task was similar to the orthographic processing test used in previous studies of Chinese reading development (e.g., Siok and Fletcher, 2001) and was comparable to the letter search task used in alphabetic languages (e.g., Mason, 1975; O’Brien et al., 2011). It was designed to test the components of visual-orthographic analysis and visual processing speed. The test materials were 100 characters selected from the dictionary and were arranged according to the visual complexity of the characters (i.e., the number of strokes within the characters). All of them were compound characters, 47 of which contained the designated component, and 81% of the selected characters were phonetic compounds that contained a semantic radical and a phonetic radical. The average number of strokes of the characters was 10 (*SD* = 3.17), and the average frequency of the characters was 227.61 per million (*SD* = 108.99)^1^. The children were presented with a series of Chinese characters arranged in a 10 × 10 matrix, and they were asked to circle the characters containing a designated component 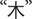 as correctly and rapidly as possible within 2 min. The score was the number of correct answers.

##### Writing fluency

In the writing fluency task, the children were asked to copy Chinese written characters from samples as correctly and rapidly as possible within 3 min. Eighty-four Chinese characters were selected from the textbooks of grades 1 to 6, fourteen characters from each grade. The average number of strokes of the selected characters was 9.6 (*SD* = 2.59). The characters were of high frequency, with an average frequency of 346.26 per million (*SD* = 799.58). There were two simple characters and eighty-two compound characters, and 76% of the compound characters were phonetic compounds. They were arranged in nine rows and ten columns according to grade from lowest to highest. The number of characters written correctly was taken as the score for writing skill.

##### Verbal working memory task

We used two digit-span tasks to measure children’s verbal working memory capacity, including a forward digit span task and a backward digit span task. In the forward digit span task, the subjects carefully listened to a sequence of digits, and they were required to repeat the digits in the same order. For example, the experimenter said, “3 4 7 5,” and the subjects needed to repeat “3 4 7 5.” In the backward digit span task, they also listened to a sequence of digits, but they were asked to repeat the digits in reverse order from the end to the beginning. For example, the experimenter said, “3 4 7 5,” and the subjects needed to repeat “5 7 4 3.” The sequences had 3 to 12 digits, and there were two items for each sequence. This task stopped when the subjects responded incorrectly in both of the two items in the same span. The score was the maximum length of span with a correct response.

##### Stroop task

The color-word Stroop task was used to examine children’s ability to inhibit interfering information. During the test, children were required to name the color of the ink and inhibit reading the word. The task demanded executive function and speed of processing, which might be related to the development of reading fluency (Leong et al., 2008). Three conditions were involved: incongruent, congruent, and neutral conditions. In the incongruent and congruent conditions, the subjects were required to name the ink color of a word, whereas the word itself was the name of a color. Interference arose in the incongruent condition when the ink color and the word meaning were incongruent [e.g., the word “
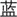
(blue)” printed in yellow ink], whereas facilitation arose in the congruent condition when the ink color and the word meaning were congruent [i.e., the word “
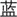
(blue)” written in blue ink]. In the neutral condition, squares were used, and the subjects were asked to name the ink color of the squares (i.e., a square printed in red ink). Each condition involved 30 printed items presented in six rows and five columns. The children were required to name the stimuli as correctly and rapidly as possible. Before each task began, there was a practice test with nine example stimuli to ensure that the children understood the task and could accurately name the colors. Time (in milliseconds) and accuracy of naming the ink color were recorded, but only the time taken for naming was analyzed. The interfering effect was determined by comparing the reaction times (RTs) in the incongruent and neutral conditions.

##### Sentence reading fluency

A reading fluency task was used to examine children’s ability to integrate word meanings into sentence comprehension on the basis of fast decoding. Relative to word reading fluency, which is often measured as the number of words correctly read within a time limit (e.g., one minute), the sentence reading fluency used in our study could better measure the multifaceted performance of both reading speed and comprehension of the text. The sentence reading fluency test was designed based on the reading fluency subtest in Woodcock-Johnson III - Tests of Achievement (Mather and Woodcock, 2001) and was similar to the reading fluency test used in previous studies of Chinese reading development (e.g., Xue et al., 2013). The test consisted of 98 sentences, which were arranged generally from short to long across the test. There was a total of 960 Chinese characters in the test, with an average of 9.8 (*SD* = 3.08) characters per sentence. The average frequency of the characters was 2385.85 per million (*SD* = 983.11). The children were asked to judge the correctness of the sentences, for example, 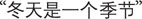
” (winter is a season), in 3 min as correctly and rapidly as possible. The score was the number of sentences that were correctly judged. To ensure that the children understood the task, two sample sentences were used for instruction and four sentences for practice before the test.

##### Word reading

A Chinese word reading test was used to assess word reading accuracy. There were 360 single-character words, of which 300 characters were selected from the textbooks of grades 1 to 6 (50 words for each grade) and 60 low-frequency words from the dictionary. The average frequency of the characters selected from textbooks was 96.12 per million (*SD* = 243.24), and the average frequency of the additional 60 low-frequency words was 0.96 per million (*SD* = 3.28). There were five simple characters and 355 compound characters, and 84% of the compound characters were phonetic compounds. These characters were presented in a sequence increasing in difficulty. The 300 characters from the textbooks were presented in sequence from the lowest to the highest grades, which were followed by the 60 low-frequency words. The children were asked to read the words aloud to the experimenters as accurately as possible. The test stopped if the children made five consecutive mistakes. The score was the number of words correctly read. The purpose of including the word reading task was to examine whether there were cognitive skills that were specifically correlated to reading fluency, as compared with their correlations with word reading accuracy.

### Results and Discussion

The means and standard deviations of the test scores are shown in Table 1. Significant differences were found between beginning and intermediate readers in reading fluency (*t* = −11.280, *p* < 0.001), word reading accuracy (*t* = −13.695, *p* < 0.001), RAN-D (*t* = 9.513, *p* < 0.001), RAN-O (*t* = 6.787, *p* < 0.001), RAN-C (*t* = 4.477, *p* < 0.001), PA (*t* = −4.592, *p* < 0.001), forward digit span (*t* = −6.368, *p* < 0.001), backward digit span (*t* = −4.175, *p* < 0.001), Chinese component search (*t* = −7.287, *p* < 0.001), and writing fluency (*t* = −17.623, *p* < 0.001), with intermediate readers showing better performance than beginning readers. There was no significant difference in inhibition effect in the Stroop task (*t* = −0.742, *p* = 0.459) or in non-verbal IQ (*t* = −1.831, *p* = 0.069) between the two groups.

There were strong correlations between reading fluency and word reading in both beginning readers (*r* = 0.751, *p* < 0.001) and intermediate readers (*r* = 0.618, *p* < 0.001). Table 2 shows the results for the correlation (after excluding the influence of age and non-verbal IQ) of measures of cognitive skills with reading fluency and word reading accuracy. We found common and differential cognitive factors that were significantly correlated with reading performance in beginning readers and intermediate readers. Specifically, for the reading fluency test, both beginning readers’ and intermediate readers’ reading fluency were significantly correlated with RAN-D (beginning readers: *r* = −0.405, *p* < 0.001; intermediate readers: *r* = −0.436, *p* < 0.001), RAN-C (beginning readers: *r* = −0.215, *p* < 0.05; intermediate readers: *r* = −0.365, *p* < 0.001), PA (beginning readers: *r* = 0.360, *p* < 0.001; intermediate readers: *r* = 0.255, *p* < 0.05), and writing fluency (beginning readers: *r* = 0.378, *p* < 0.001; intermediate readers: *r* = 0.363, *p* < 0.001). In addition, intermediate readers’ performance was also significantly correlated with RAN-O (*r* = −0.447, *p* < 0.001). For the word reading accuracy test, beginning readers’ performance were significantly correlated with RAN-D (*r* = −0.354, *p* < 0.001) and PA (*r* = 0.369, *p* < 0.001), while intermediate readers’ performance of word reading was significantly correlated with RAN-D (*r* = −0.357, *p* < 0.001), RAN-O (*r* = −0.379, *p* < 0.001), RAN-C (*r* = −0.261, *p* < 0.05), and forward digit span (*r* = 0.215, *p* < 0.05). Thus, these results indicated that RAN-D correlated with both reading fluency and word reading in both age groups, whereas RAN-O and RAN-C had larger correlations with reading performance in intermediate readers than in beginning readers. The correlations between PA and reading performance (both reading fluency and word reading accuracy) decreased from beginning readers to intermediate readers. Moreover, writing fluency was more specifically correlated with reading fluency but not word reading accuracy across age groups.

**TABLE 2:** Partial correlations between reading performance and component skills, controlling for age and non-verbal intelligence.

	**Beginner**		**Intermediate**	
	
	**Reading fluency**	**Word reading**	**Reading fluency**	**Word reading**
RAN-D	−0.405***	−0.354***	−0.436***	−0.357***
RAN-O	–0.170	–0.103	−0.447***	−0.379***
RAN-C	−0.215*	–0.145	−0.365***	−0.261*
PA	0.360***	0.369***	0.255*	0.191
Forward digit span	0.112	0.147	0.090	0.215*
Backward digit span	0.174	0.189	0.123	0.192
Stroop	0.150	0.201	–0.184	–0.044
Chinese component search	0.044	0.004	0.107	0.148
Writing	0.378***	0.184	0.363***	0.164

Asterisk(s) indicates significance levels: *p < 0.05; **p < 0.01; and ***p < 0.001.

To determine the relative power of these cognitive factors for predicting sentence reading fluency and word reading accuracy, we carried out a series of fixed order hierarchical multiple regression analyses. The results are shown in Table 3-1. For reading fluency, RAN, PA, and writing were the three most powerful predictors for both beginning readers and intermediate readers. Specifically, for beginning readers, RAN-D [8.3% of variance (Δ*F* = 17.120, *p* < 0.001)], RAN-C [2.3% of variance (Δ*F* = 4.216, *p* = 0.043)], PA [6.5% of variance (Δ*F* = 12.930, *p* < 0.001)], and writing [7.2% of variance (Δ*F* = 14.525, *p* < 0.001)] were the significant predicters for reading fluency. For intermediate readers, RAN-D [13.3% of variance (Δ*F* = 19.675, *p* < 0.001)], RAN-O [14% of variance (Δ*F* = 20.938, *p* < 0.001)], RAN-C [9.3% of variance (Δ*F* = 12.875, *p* < 0.001)], PA [4.6% of variance (Δ*F* = 5.839, *p* = 0.018)], and writing [9.3% of variance (Δ*F* = 12.751, *p* < 0.001)] were the significant predictors for reading fluency. The results indicated that the predictive power of RAN and writing fluency increased from beginning to intermediate readers, whereas the predictive power of PA diminished.

**TABLE 3-1:** Summary of hierarchical multiple regressions that tested the predictive power of various component skill measures for reading fluency.

		**Reading fluency (**Δ***R*^2^)**	
**Step**	**Predictor**	**Beginner**	**Intermediate**
*Controlling for variation in age and non-verbal IQ*			
1	age	0.431***	0.071*
2	IQ	0.065***	0.226***
3	RAN-D	0.083***	0.133***
3	RAN-O	0.015	0.140***
3	RAN-C	0.023*	0.093***
3	PA	0.065***	0.046*
3	Forward digit span	0.006	0.006
3	Backward digit span	0.015	0.011
3	Stroop	0.011	0.024
3	Chinese component search	0.001	0.008
3	Writing	0.072***	0.093***

Asterisk(s) indicates significance levels: *p < 0.05, **p < 0.01, and ***p < 0.001.

As revealed in the above regression analyses, RAN, PA, and writing fluency were the three most powerful predictors of reading fluency. Among these three factors, RAN and PA may share common variance of phonological process (Liao et al., 2007), whereas RAN and writing fluency may share common variance reflecting general speeded processing. To estimate the unique predictive power of these three factors, we performed another set of fixed-order multiple regression analysis. The results are shown in Table 3-2. First, to determine the contribution of phonological awareness and writing after RAN was controlled, children’s age and IQ were again entered into the model as steps 1 and 2, and RAN was entered as steps 3–5. PA and writing were then entered as step 6. The results showed that, in the beginning readers, PA accounted for 4% (Δ*F* = 8.991, *p* < 0.01) of the variance, whereas writing accounted for 3.2% (Δ*F* = 6.941, *p* < 0.01) of the variance. In the intermediate readers, PA and writing did not account for additional variance in reading performance. The results suggested that in the beginning readers, PA and writing contributed to reading fluency even after the effect of RAN was partialled out, but in the intermediate readers, PA and writing did not account for additional variance in reading fluency.

**TABLE 3-2:** Summary of hierarchical multiple regressions that tested the predictive power of various component skill measures for reading fluency.

**Predicators**	**Beginner**		**Intermediate**	
	***R*^2^**	***R*^2^ change**	***R*^2^**	***R*^2^ change**
(1) Age	0.431	0.431***	0.071	0.071*
(2) Non-verbal IQ	0.495	0.065***	0.279	0.226***
(3) RAN-D	0.578	0.083***	0.431	0.133***
(4) RAN-O	0.578	0.000	0.466	0.035*
(5) RAN-C	0.582	0.003	0.475	0.009
(6) PA	0.622	0.040**	0.477	0.002
(1) Age	0.431	0.431***	0.071	0.071*
(2) Non-verbal IQ	0.495	0.065***	0.279	0.226***
(3) RAN-D	0.578	0.083***	0.431	0.133***
(4) RAN-O	0.578	0.000	0.466	0.035*
(5) RAN-C	0.582	0.003	0.475	0.009
(6) Writing	0.614	0.032**	0.499	0.024
(1) Age	0.431	0.431***	0.071	0.071*
(2) Non-verbal IQ	0.495	0.065***	0.279	0.226***
(3) RAN-D	0.578	0.083***	0.431	0.133***
(4) RAN-O	0.578	0.000	0.466	0.035*
(5) RAN-C	0.582	0.003	0.475	0.009
(6) Writing	0.614	0.032**	0.499	0.024
(7) PA	0.636	0.022*	0.503	0.004
(1) Age	0.431	0.431***	0.071	0.071*
(2) Non-verbal IQ	0.495	0.065***	0.279	0.226***
(3) RAN-D	0.578	0.083***	0.431	0.133***
(4) RAN-O	0.578	0.000	0.466	0.035*
(5) RAN-C	0.582	0.003	0.475	0.009
(6) PA	0.622	0.040**	0.477	0.002
(7) Writing	0.636	0.013	0.503	0.026*
(1) Age	0.431	0.431***	0.071	0.071*
(2) Non-verbal IQ	0.495	0.065***	0.279	0.226***
(3) PA	0.561	0.065***	0.343	0.046*
(4) Writing	0.591	0.030*	0.428	0.085***
(5) RAN-D	0.623	0.032**	0.466	0.038*
(1) Age	0.431	0.431***	0.071	0.071*
(2) Non-verbal IQ	0.495	0.065***	0.279	0.226***
(3) PA	0.561	0.065***	0.343	0.046*
(4) Writing	0.591	0.030*	0.428	0.085***
(5) RAN-O	0.593	0.002	0.485	0.057**
(1) Age	0.431	0.431***	0.071	0.071*
(2) Non-verbal IQ	0.495	0.065***	0.279	0.226***
(3) PA	0.561	0.065***	0.343	0.046*
(4) Writing	0.591	0.030*	0.428	0.085***
(5) RAN-C	0.614	0.023*	0.471	0.043*

The number on the left indicates the predicators’ order of entering the equation. *p < 0.05, **p < 0.01, and ***p < 0.001.

Second, to further estimate the unique predictive power of PA and writing for reading fluency, we entered PA or writing as step 7 in the model after entering all the other related factors (Table 3-2). We found that, in the beginning readers, PA still explained a significant proportion of the variance [2.2%, (Δ*F* = 4.983, *p* = 0.028)] in reading fluency after the effects of RAN and writing were controlled, but the effect of writing was not significant after the effects of RAN and PA were controlled. In contrast, in the intermediate readers, writing explained 2.6% (Δ*F* = 4.192, *p* = 0.044) of the variance in reading fluency after the effect of RAN and PA were controlled, but PA did not account for the additional variance of reading fluency. We then entered subtests of RAN as step 5 in the model after the effects of PA and writing were controlled (Table 3-2). We found that RAN-D [3.2%, (Δ*F* = 7.271, *p* < 0.01)] and RAN-C [2.3%, (Δ*F* = 5.003, *p* = 0.028)] explained significant proportions of variance in reading fluency in beginning readers, and RAN-D [3.8%, (Δ*F* = 5.881, *p* = 0.018)], RAN-O [5.7%, (Δ*F* = 9.035, *p* < 0.01)], and RAN-C [4.3%, (Δ*F* = 6.736, *p* = 0.011)] explained significant proportions of variance in intermediate readers, suggesting that RAN made a unique contribution to reading fluency that cannot be explained by PA or writing fluency. The results indicated that, among the three most powerful predictors of reading fluency (i.e., RAN, PA, and writing fluency), RAN made a unique contribution to reading fluency that cannot be explained by PA or writing fluency in both beginning and intermediate readers and that the predictive power of RAN increased as age increased. In contrast, PA accounted for unique variance in reading fluency only in beginning readers. The effect of writing fluency on reading fluency was strong in both age groups, but it seemed to share considerable common variance with RAN.

Furthermore, to compare the predictive powers of the cognitive factors for reading fluency with those for word reading accuracy, we performed similar regression analyses for the word reading task. The results are shown in Tables 4-1, 4-2. For beginning readers, RAN-D and PA were the most significant predictors for their word reading ability, accounting for 6.8% (Δ*F* = 12.502, *p* ≤ 0.01) and 7.4% of variance (Δ*F* = 13.729, *p* < 0.001), respectively. For intermediate readers, RAN-D [10.1% of variance (Δ*F* = 12.247, *p* < 0.01)], RAN-O [11.4% of variance (Δ*F* = 14.089, *p* < 0.001)], RAN-C [5.4% of variance (Δ*F* = 6.116, *p* = 0.015)], and forward digit span [3.7% of variance (Δ*F* = 4.074, *p* = 0.047)] were the significant predictors for word reading ability (Table 4-1). The results indicated that the effect of RAN on Chinese word reading increased whereas the effect of PA decreased from beginning to intermediate readers. Moreover, in the intermediate readers, forward digit span also accounted for a significant proportion of variance in word reading, but it did not make a significant contribution to reading fluency.

**TABLE 4-1:** Summary of hierarchical multiple regressions that tested the predictive power of various component skill measures for Chinese word reading.

		**Chinese word reading (**Δ***R*^2^)**	
**Step**	**Predictors**	**Beginner**	**Intermediate**
*Controlling for variation in age and non-verbal IQ*			
1	Age	0.388***	0.081**
2	IQ	0.068***	0.125***
3	RAN-D	0.068**	0.101**
3	RAN-O	0.006	0.114***
3	RAN-C	0.012	0.054*
3	PA	0.074***	0.029
3	Forward digit span	0.012	0.037*
3	Backward digit span	0.019	0.029
3	Stroop	0.022	0.002
3	Chinese component search	0.000	0.017
3	Writing	0.018	0.021

Asterisk(s) indicates significance levels: *p < 0.05, **p < 0.01, and ***p < 0.001.

**TABLE 4-2:** Summary of hierarchical multiple regressions that tested the predictive power of various component skill measures for Chinese word reading.

**Predicators**	**Beginner**		**Intermediate**	
	***R*^2^**	***R*^2^ change**	***R*^2^**	***R*^2^ change**
(1) Age	0.388	0.388***	0.081	0.081**
(2) Non-verbal IQ	0.456	0.068***	0.206	0.125***
(3) RAN-D	0.524	0.068***	0.307	0.101***
(4) RAN-O	0.526	0.002	0.339	0.032*
(5) RAN-C	0.527	0.001	0.340	0.001
(6) PA	0.578	0.051**	0.340	0.000
(1) Age	0.388	0.388***	0.081	0.081**
(2) Non-verbal IQ	0.456	0.068***	0.206	0.125***
(3) RAN-D	0.524	0.068***	0.307	0.101***
(4) RAN-O	0.526	0.002	0.339	0.032*
(5) RAN-C	0.527	0.001	0.340	0.001
(6) Forward digit span	0.535	0.007	0.351	0.011
(1) Age	0.388	0.388***	0.081	0.081**
(2) Non-verbal IQ	0.456	0.068***	0.206	0.125***
(3) RAN-D	0.524	0.068***	0.307	0.101***
(4) RAN-O	0.526	0.002	0.339	0.032*
(5) RAN-C	0.527	0.001	0.340	0.001
(6) Forward digit span	0.535	0.007	0.351	0.011
(7) PA	0.584	0.049**	0.351	0.001
(1) Age	0.388	0.388	0.081	0.081**
(2) Non-verbal IQ	0.456	0.068	0.206	0.125***
(3) PA	0.530	0.074	0.235	0.029
(4) Forward digit span	0.538	0.008	0.267	0.032
(5) RAN-D	0.575	0.036**	0.322	0.055*
(1) Age	0.388	0.388	0.081	0.081**
(2) Non-verbal IQ	0.456	0.068	0.206	0.125***
(3) PA	0.530	0.074	0.235	0.029
(4) Forward digit span	0.538	0.008	0.267	0.032
(5) RAN-O	0.539	0.000	0.338	0.071**
(1) Age	0.388	0.388	0.081	0.081**
(2) Non-verbal IQ	0.456	0.068	0.206	0.125***
(3) PA	0.530	0.074	0.235	0.029
(4). Forward digit span	0.538	0.008	0.267	0.032
(5) RAN-C	0.550	0.012	0.301	0.034*

The number on the left indicates the predicators’ order of entering the equation. *p < 0.05, **p < 0.01, and ***p < 0.001.

To determine the contribution of PA (and also forward digit span) after the effect of RAN was controlled, we performed fixed-order multiple regression analyses with children’s age and IQ being again entered into the model as steps 1 and 2, and RAN being entered as steps 3–5. PA or forward digit span was then entered as step 6 (Table 4-2). In beginning readers, PA accounted for 5.1% (Δ*F* = 10.067, *p* < 0.01) of the variance, whereas forward digit span did not account for additional variance in word reading performance. In intermediate readers, PA and forward digit span did not account for additional variance in word reading performance. The results suggested that PA played a more important role in word reading accuracy in the beginning readers than in the intermediate readers.

To evaluate the unique predictive power of PA and RAN for word reading accuracy, we entered PA or RAN as the last step in the model after entering all of the other factors (Table 4-2). We found that PA still contributed 4.9% (Δ*F* = 9.765, *p* < 0.01) to the variance in word reading in beginning readers. In addition, only RAN-D explained 3.6% (Δ*F* = 7.255, *p* < 0.01) of variance in word reading for beginning readers. For intermediate readers, RAN-D [5.5% of variance (Δ*F* = 6.614, *p* = 0.012)], RAN-O [7.1% of variance (Δ*F* = 8.758, *p* < 0.01)], and RAN-C [3.4% of variance (Δ*F* = 3.964, *p* = 0.050)] explained significant proportions of variance in word reading. The results suggested that there were strong effects of RAN on word reading accuracy across beginning and intermediate readers, and the findings were consistent with those in reading fluency.

In summary, we identified cognitive factors that contributed significantly to reading fluency, including RAN (particularly rapid naming of digits), PA, and writing fluency. We found that rapid naming of digits consistently accounted for the unique variance both of reading fluency and word reading accuracy and across age groups, indicating that rapid digit naming may play a more important role in reading development than rapid naming of objects or colors. The results were consistent with previous findings. For example, Georgiou et al. (2008) found that rapid digit naming had greater correlations with reading performance than color naming in English and Greek. The findings indicated that it was not simply the speeded processing component that was important for reading fluency. Rapid digit naming, as an alphanumeric RAN task, may better capture the underlying phonological components of reading abilities that do the non-alphanumeric RAN tasks of rapid object and color naming. In addition, PA accounted for the unique variance of reading fluency and reading accuracy only in beginning readers but not in intermediate readers, indicating that the role of PA in Chinese reading abilities may decrease during reading development. While RAN and PA have been consistently demonstrated to contribute to reading development in alphabetic languages, the effect of writing skills has been inconsistent and small (Guan et al., 2011). The importance of writing fluency in Chinese reading may be more related to the inherent properties of Chinese writing systems (Tan et al., 2005). Our study demonstrated that writing fluency was increasingly important in predicting reading fluency during development and that it made a significant contribution to reading fluency but not to word reading accuracy, suggesting that it was more specifically correlated with reading rate.

## Study 2: the Effect of Visual Crowding on Chinese Reading Fluency

The multiple factors that contributed to reading fluency in Chinese children have highlighted the components of phonology, speeded processing, visual-motor process, and so on. In Study 2, we focused on visual crowding and explored whether this visual factor accounted for the unique variance of Chinese reading fluency. Previous studies have shown that visual crowding, a key factor in how people visually recognize objects, can severely affect reading speed (Pelli, 2008). Individuals with reading disabilities also show greater visual crowding effects, which limits word identification in multi-item processing (Martelli et al., 2009). In this study, we examined the role of visual crowding in Chinese reading fluency and tested whether it contributed unique variance to Chinese reading fluency even after the effects of other cognitive skills (such as RAN, PA, and writing fluency) were controlled.

### Methods

#### Participants

Ninety children participated in this study. They were 2nd and 3rd graders from Study 1. Data for four participants were excluded because they failed to complete the visual crowding test. The final sample included 86 children (52 males and 34 females) with a mean age of 8.5 years (*SD* = 0.63). All subjects had normal or corrected-to-normal vision. Written consent was obtained from their parents before the tests. They were assessed with the following tests besides the visual crowding test: RAN-D [*mean* (*M*) = 25.39, *SD* = 5.5], RAN-O (*M* = 24.73, *SD* = 4.5), RAN-C (*M* = 30.01, *SD* = 8.0), PA (*M* = 12.99, *SD* = 5.1), forward digit span (*M* = 7.20, *SD* = 1.0), backward digit span (*M* = 3.64, *SD* = 1.4), Stroop task (*M* = 53.47, *SD* = 11.3), Chinese component search (*M* = 28.87, *SD* = 6.6), writing fluency (*M* = 40.01, *SD* = 7.9), non-verbal IQ (*M* = 66.63, *SD* = 25.1), Chinese word reading (*M* = 116.33, *SD* = 40.7), and reading fluency test (*M* = 31.63, *SD* = 12.5).

#### Stimuli and Apparatus

**FIGURE 1:**
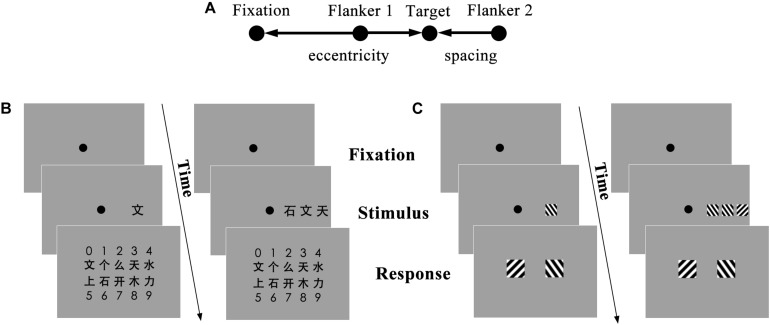
The procedure of the visual crowding test. **(A)** Definition of the typical parameters for crowding. Critical spacing is the center-to-center distance between the target and flanker, and eccentricity is the distance between the target and fixation. **(B)** In the uncrowded condition (left) of the Chinese characters (VCC) test, only a single character (the target) is presented. In the crowded condition (right), three characters (the target is in the middle) are presented. During the response period, the subject chooses the target from 10 Chinese characters by pressing a key. **(C)** The procedure of the visual crowding test for gratings (VCG). The uncrowded gratings condition is on the left, and the crowded condition is in the right. The fixation point is in the center of the screen.

In the visual crowding test, stimuli were displayed using Matlab 2017a with the Psychtoolbox extension (Brainard, 1997) against a gray background running on a Dell Precision 3510 computer, which had a 15.6-in. Intel (R) HD Graphics 530 screen (frame rate of 59 Hz, resolution of 1920 × 1080 pixels). We used Chinese characters (VCC) and visual gratings (VCG) as stimuli. The gratings were visual stimuli in the absence of any linguistic information, and they have been used in many previous studies (Pelli, 2008). We used the visual gratings test as a control condition to examine whether the correlation, if any, between crowding effect and reading fluency was related specifically to linguistic stimuli (VCC) or more generally to both linguistic and non-linguistic visual crowding. The test consisted of uncrowded and crowded conditions. In the VCC test, the uncrowded condition had only one Chinese character, which was displayed randomly as a target on the left or right side of the fixation point. Previous studies have revealed that crowding depended on the ratio of spacing (center-to-center distance between the target and flanker) and eccentricity (the distance between the target and fixation point) (Figure 1A). A ratio of less than 0.5 could produce crowding, and the strength of the crowding effect increased as the ratio decreased (Bouma, 1970). In order to produce crowding without stimulus overlap, the stimulus in the crowded condition was a three-character string of equally sized, equally spaced Chinese characters arranged in a horizontal orientation, with spacing of 2° and eccentricity of 5°, resulting in a ratio of 0.4. The characters presented at the same time were randomly selected without repetition from a group of 10 characters that were selected from the first-grade Chinese textbooks and had 3–5 strokes. The font type bold Heiti (black font) was used because of its relatively uniform stroke width, and the Chinese characters had the same width and height (width: 1°). The visual crowding between gratings (VCG) test was similar to the VCC test, with the Chinese characters in the VCC test replaced by a square patch of a sinusoidal grating (length and width: 1° and 1°; contrast: 1; orientation: ±45°). Each trial started with a 1° fixation point, which was presented for 1000 ms at the center of the screen. In the VCC and VCG conditions, the stimulus of a Chinese character or grating, respectively, was displayed for 250 ms.

#### Procedure

The tests were conducted in a quiet and dimly lit room. During the test, the participants sat 45 cm from the screen of the computer, and they were required to focus their eyes on a fixation point while observing the target with their peripheral vision. The visual crowing test included two runs (VCC and VCG). Each run contained two conditions (i.e., the crowded and uncrowded conditions), and each condition included 60 trials. There were five practice trials before the formal test to make sure that the participants understood the task. In the uncrowded condition, a Chinese character or a grating (the target) appeared randomly to the left or right of the fixation point. The subjects were asked to choose the target from 10 Chinese characters or from two gratings. We only offered the children two options because it was difficult for the young children to identify subtle differences in the orientation of the grating if many options were provided. In the crowded condition, the target was surrounded by two other flankers horizontally, and the subjects were asked to ignore the two flankers and pay attention to the target (Figures 1B,C). They were then asked to identify the target by pressing the keyboard, i.e., the numeric keys “0–9” corresponding to different Chinese characters and “F” and “J” corresponding to grating orientation (left and right). Response accuracy and reaction time were recorded.

### Results and Discussion

Performance accuracy has been shown to be a reliable behavior indicator of crowding (Whitney and Levi, 2011). Therefore, the following results are based on the accuracy of the subjects’ response: uncrowded VCC [*mean accuracy* (*MA*) = 0.87, SD = 0.14], crowded VCC (*MA* = 0.73, *SD* = 0.21), uncrowded VCG (*MA* = 0.72, *SD* = 0.17), and crowded VCG (*MA* = 0.67, *SD* = 0.14). We first conducted a paired *t*-test between crowded and uncrowded conditions and found significant crowding effects for both word condition (*t* = 7.879, *p* = 0.000) and grating condition (*t* = 4.110, *p* = 0.000). We then calculated the visual crowding strength (VCS), which reflected the extent to which the subjects’ performance was affected by crowding, using the following equation (Gong et al., 2018) (1):

(1) $$\text{VCS}=\,\frac{U\,n\,c\,r\,o\,w\,d\,e\,d-C\,r\,o\,w\,d\,e\,d}{U\,n\,c\,r\,o\,w\,d\,e\,d}$$

where the numerator is the difference in the accuracy between the uncrowded and crowded condition, and the denominator is the accuracy in the uncrowded condition, which serves as the baseline. The mean visual crowding strength was 0.17 (*SD* = 0.21) for Chinese characters (VCSC) and 0.05 (*SD* = 0.18) for gratings (VCSG).

We then tested the correlation between reading performance and the crowding effect. VCSC was significantly correlated with reading fluency after the effect of age and non-verbal IQ was controlled. In contrast, VCSG was not related to reading performance or any other cognitive skills (Table 5), suggesting that reading fluency may be more specifically affected by the word-induced crowding effect. In addition, VCSC could not explain significant variance of Chinese word reading accuracy [VCSC (Δ*F* = 1.145, *p* = 0.29)]. The greater impact of visual crowding on sentence reading fluency relative to word reading accuracy may be due to the fact that children’s reading fluency was tested with characters presented in closely spaced continuous lists (Jones et al., 2009).

**TABLE 5:** Partial correlations between visual crowding effect and reading performance and cognitive skills, controlling for age and non-verbal intelligence.

	**VCSG**	**VCSC**
Reading fluency	–0.140	−0.260*
Word reading	0.016	–0.117
RAN-D, sec	0.175	0.026
RAN-O, sec	0.145	0.035
RAN-C, sec	–0.012	–0.083
PA	–0.118	–0.126
Forward digit span	0.115	–0.105
Backward digit span	–0.066	0.149
Stroop	0.030	0.108
Chinese component search	–0.063	–0.203
Writing	–0.095	–0.027

VCSG, visual crowding strength of gratings; VCSC, visual crowding strength of Chinese characters. Asterisk(s) indicates significance levels: *p < 0.05 and **p < 0.01.

To further examine the contribution of visual crowding to reading fluency relative to the other cognitive skills, we conducted a series of fixed-order hierarchical multiple regressions, with children’s age and IQ being entered into the model as steps 1 and 2, and other skills, including VCSC, RAN, PA, and writing, as step 3 (Table 6-1). We compared the relative contribution of VCSC with those of RAN, PA, and writing because these three factors were found to be critical in predicting reading fluency, as revealed in Study 1. The results showed that VCSC explained 4.4% (Δ*F* = 5.949, *p* = 0.017) of variance in reading fluency. In addition, RAN-D, RAN-O, RAN-C, and writing fluency explained 15.0% (Δ*F* = 24.667, *p* < 0.001), 3.3% (Δ*F* = 4.432, *p* < 0.05), 3.9% (Δ*F* = 5.305, *p* < 0.05), and 8.8% (Δ*F* = 12.826, *p* = 0.001) of the variance, respectively. In contrast, PA could not explain a significant proportion of variance in reading fluency (Δ*F* = 3.313, *p* = 0.072).

**TABLE 6-1:** Summary of hierarchical multiple regressions that tested the predictive power of various component skill measures for reading fluency.

**Step**	**Predictors**	**Reading fluency (**Δ***R*^2^)**
*Controlling for variation in age and non-verbal IQ*		
1	Age	0.120**
2	IQ	0.231***
3	VCSC	0.044*
3	VCSG	0.013
3	RAN-D	0.15***
3	RAN-O	0.033*
3	RAN-C	0.039*
3	PA	0.025
3	Forward digit span	0.01
3	Backward digit span	0.006
3	Stroop	0.022
3	Chinese component search	0.003
3	Writing	0.088**

Asterisk(s) indicates significance levels: *p < 0.05; **p < 0.01; and ***p < 0.001.

Furthermore, we tested whether VCSC, RAN, and writing fluency accounted for the unique variance of reading fluency. The results showed that after the effects of RAN, PA, and writing were controlled, VCSC still accounted for 4.5% (Δ*F* = 8.369, *p* < 0.01) of variance (Table 6-2), suggesting that VCSC was a powerful predictor of reading fluency and accounted for unique variance that was independent of PA, writing, and RAN. Writing was also a powerful predictor of reading fluency and explained 3.4% of variance (Δ*F* = 6.400, *p* = 0.013) after the effects of VCSC, RAN, and PA were controlled. In addition, after VCSC, writing and PA were controlled, RAN-D and RAN-C were significant predictors, which contributed 7.5% (Δ*F* = 13.935, *p* < 0.001) and 3.8% (Δ*F* = 6.472, *p* < 0.05) of the variance in reading fluency, respectively. The results suggested that visual crowding of characters accounted for unique variance of reading fluency in children that could not be explained by RAN, PA, or writing fluency.

**TABLE 6-2:** Summary of hierarchical multiple regressions that tested the predictive power of visual crowding effects for reading fluency.

**Step**	**Predictors**	**Reading fluency (**Δ***R*^2^)**
Controlling for variation in age, non-verbal IQ, RAN, PA, and Writing		
1	Age	0.120**
2	Non-verbal IQ	0.231***
3	RAN-D	0.150***
4	RAN-O	0.000
5	RAN-C	0.006
6	PA	0.007
7	Writing	0.030*
8	VCSC	0.045**
Controlling for variation in age, non-verbal IQ, VCSC, RAN, and PA		
1	Age	0.120**
2	Non-verbal IQ	0.231***
3	VCSC	0.044*
4	RAN-D	0.146***
5	RAN-O	0.000
6	RAN-C	0.010
7	PA	0.003
8	Writing	0.034*
Controlling for variation in age, non-verbal IQ, VCSC, Writing, and PA		
1	Age	0.120**
2	Non-verbal IQ	0.231***
3	VCSC	0.044*
4	Writing	0.091***
5	PA	0.011
6	RAN-D	0.075***
6	RAN-O	0.010
6	RAN-C	0.038*

The number on the left indicates the predicators’ order of entering the equation. Asterisk(s) indicates significance levels: *p < 0.05, **p < 0.01 and ***p < 0.001.

## General Discussion

The current study extended previous findings and tested which cognitive factors accounted for individual differences in reading fluency in logographic Chinese. According to Perfetti’s verbal efficiency model, reading efficiency is affected by both lexical processes and cognitive capacities. Our results generally indicated a greater importance for lexical cognitive factors (including PA, RAN, and writing fluency) in Chinese reading fluency than for cognitive capacities such as working memory and executive control. We further demonstrated in Study 2 that visual crowding accounted for unique variance in reading fluency and that the effect was much more pronounced for crowding caused by linguistic stimuli than for gratings. The impact of visual crowding may be related to the presentation format, in that the characters were presented in close-spaced continuous lists during reading.

The results showed that children’s reading fluency was affected mainly by RAN, writing fluency, and PA but that the roles of these factors were different in beginning readers and intermediate readers. We found that RAN was a strong predictor of both reading fluency and word reading accuracy and that the predictive power was strong in both beginning readers and intermediate readers. In contrast, PA seemed to be more important for reading fluency and reading accuracy for beginning readers than for intermediate readers. Writing fluency predicted reading fluency but not word reading accuracy, and it played an increasingly important role during reading development. The effect of writing skill may reflect accommodation to the demands of learning to read Chinese.

The importance of RAN in reading fluency has been well-established across alphabetic orthographies (Protopapas et al., 2013), and deficit of RAN has been frequently reported in dyslexic readers (Denckla and Rudel, 1976; Wolf et al., 1986; van den Bos, 1998). In line with previous studies (Liao et al., 2007; Liu et al., 2017), we found that RAN was a particularly strong predictor of the reading fluency of Chinese and that its influence was strong across beginning and intermediate readers. Besides, RAN, particularly rapid naming of digits, was also closely correlated to word reading accuracy, although it accounted for less variance in word reading accuracy than in reading fluency, suggesting that the relationship of RAN and reading fluency was driven not simply by a general speeded process but by processes that were related to the connections between visual and phonological process (Norton and Wolf, 2012). One explanation for the important role of RAN skills in predicting reading fluency was that it involved connecting and automatizing the visual stimuli with their linguistic information, which was similar to the process of reading (Denckla and Rudel, 1976; Norton and Wolf, 2012).

Phonological awareness contributed to Chinese reading fluency in our study, but the effect diminished from beginning to intermediate readers. In addition, the predictive power of PA for word-level reading accuracy was significant in beginning readers but not in intermediate readers. The results were consistent with previous studies (Huang and Hanley, 1995; Xue et al., 2013). For example, Georgiou et al. (2008) found that the impact of PA on reading correlated with reading fluency during early reading acquisition (Georgiou et al., 2008). Moreover, phonological processing has been reported to contribute to reading abilities in different countries, but the role of different levels of phonological awareness may vary across cultures (McBride-Chang et al., 2004; Ziegler and Goswami, 2005). Whereas phoneme awareness was a better predictor of early reading skills than onset/rime awareness in alphabetic languages (Hulme et al., 2002), onset/rime awareness was much more important than phoneme awareness in the development of Chinese reading (Siok and Fletcher, 2001). In our study, we focused on PA at the onset-rime level. The results extended previous studies and demonstrated the important role of onset-rime PA in reading fluency.

While there have been a great number of studies investigating RAN and reading fluency, studies examining the relationship between writing fluency and reading fluency are limited. Previous studies found that the effect of writing on reading was less robust and inconsistent in alphabetic systems, but research on Chinese had demonstrated writing to have a beneficial effect on reading development (Tan et al., 2005; Guan et al., 2011; Van Yip Tso et al., 2012). For example, Tan et al. (2005) found a close correlation between children’s ability to copy characters and word reading fluency. The relationship was even greater than the relationship between PA and reading. Consistent with these studies, we found that writing fluency was closely related to reading fluency of Chinese in both beginning and intermediate readers. In addition, writing was more specifically related to reading fluency than word-reading accuracy. Writing practice may facilitate the orthographic representation of Chinese characters and strengthen the link from orthography to meaning (Tan et al., 2005; Guan et al., 2011), which helps words to be recognized efficiently and automatically. Previous neuroimaging studies have supported this notion and showed that writing characters established higher quality orthographic representation, which was associated with greater activation in bilateral superior parietal lobules and lingual gyri (Cao et al., 2013).

Moreover, we found that children who were more affected by visual crowding also performed worse in the reading fluency test. Visual crowding contributed unique variance to reading fluency of Chinese after the effects of RAN, PA, and writing fluency had been controlled. Despite crowding being well-known as an essential bottleneck to reading in the periphery, there has previously been a dearth of research into what the role of crowding is in reading development relative to other cognitive factors. During Chinese reading, it was found that there was both within-character (predominantly in complex characters) and between-characters crowding, with the latter being much stronger (Zhang et al., 2009). The strong between-characters crowding effect may result from improper integration of visual features of target and flanker characters in the periphery (Levi et al., 2002; Pelli et al., 2004).

Our findings have important implications for the potential treatment for individuals who suffer from reading disabilities, particularly in terms of reading fluency. Previous studies demonstrated that intensive instruction in phonological skills improved children’s decoding and word identification but provided only minimal gains in reading fluency (Meyer and Felton, 1999; Norton and Wolf, 2012), whereas training programs that targeted most components of reading significantly improved both reading accuracy and fluency (Wolf et al., 2009; Morris et al., 2012). In this study, we found that RAN, PA, writing, and visual crowding played important roles in reading fluency of Chinese. A combination of training approaches focusing on these factors may help Chinese children develop more automatic reading and have beneficial intervention effects on children who struggle with reading fluency.

Finally, there are some limitations to this study that are worth mentioning. First, in the Stroop task, we used squares instead of words that were unrelated to color in the neutral condition. The difference in visual complexity (squares vs. words) between the neutral and incongruent conditions may influence the differential time that we used as an index of the interference effect. Second, for the visual crowding test, different numbers of options were provided to the children in the VCC and VCG conditions. Although we did not directly compare between the two conditions and instead conducted correlational analyses between reading performance and crowding effect separately for the VCC and VCG conditions, this resulted in incomparable baseline accuracy for the VCC and VCG conditions and affected the values of visual crowding strength. Future studies are needed to overcome these limitations and to employ more sophisticated manipulation of the spatial and temporal relations between the targets and flankers to better characterize and understand the influence of the crowding effect on reading and particularly to identify at which stages of reading the crowding has its effect. Third, the data presented in our study were cross-sectional and correlational, so we could not make any causal assertions. Further studies using a longitudinal design are essential to further explore the causal effects of cognitive factors on reading fluency in Chinese children.

## Data Availability Statement

The datasets generated for this study are available on request to the corresponding author.

## Ethics Statement

The studies involving human participants were reviewed and approved by Institute of Psychology Chinese Academy of Sciences. Written informed consent to participate in this study was provided by the participants’ legal guardian/next of kin.

## Author Contributions

JB, WL, YY, WH, and MX designed the research. JB, WL, WH, and MX performed the research and analyzed the data. All authors wrote the manuscript.

## Conflict of Interest

The authors declare that the research was conducted in the absence of any commercial or financial relationships that could be construed as a potential conflict of interest.

## Footnotes

1 www.cncorpus.org

## References

[B1] Baddeley A. (2003). Working memory and language: an overview. *J. Commun. Disord.* 36 189–208. 12742667 10.1016/s0021-9924(03)00019-4

[B2] Bouma H. (1970). Interaction effects in parafoveal letter recognition. *Nature* 226 177–178. 10.1038/226177a0 5437004

[B3] Brainard D. H. (1997). The psychophysics toolbox. *Spat. Vis.* 10 433–436. 10.1163/156856897x00357 9176952

[B4] Breznitz Z. (2006). *Fluency in Reading: Synchronization of Processes.* Mahwah, NJ: Erlbaum.

[B5] Cao F. Vu M. Chan D. H. L. Lawrence J. M. Harris L. N. Guan Q. (2013). Writing affects the brain network of reading in Chinese: a functional magnetic resonance imaging study. *Hum. Brain Mapp.* 34 1670–1684. 10.1002/hbm.22017 22378588 PMC6870511

[B6] Cheung L. Y. T. Cheung S.-H. (2017). Chinese-character crowding-I. Effects of structural similarity. *J. Vis.* 17:14. 10.1167/17.11.14 28973567

[B7] Chung K. K. McBride-Chang C. Wong S. W. Cheung H. Penney T. B. Ho C. S. (2008). The role of visual and auditory temporal processing for Chinese children with developmental dyslexia. *Ann. Dyslexia* 58 15–35. 10.1007/s11881-008-0015-4 18483866

[B8] Denckla M. B. Rudel R. G. (1976). Naming of object-drawings by dyslexic and other learning disabled children. *Brain Lang.* 3 1–15. 773516 10.1016/0093-934x(76)90001-8

[B9] Ehri L. C. (2013). Orthographic mapping in the acquisition of sight word reading, spelling memory, and vocabulary learning. *Sci. Stud. Read.* 18 5–21. 10.1080/10888438.2013.819356

[B10] Elhassan Z. Crewther S. G. Bavin E. L. (2017). The contribution of phonological awareness to reading fluency and its individual sub-skills in readers aged 9- to 12-years. *Front. Psychol.* 8:533. 10.3389/fpsyg.2017.00533 28443048 PMC5387103

[B11] Georgiou G. K. Parrila R. Papadopoulos T. C. (2008). Predictors of word decoding and reading fluency across languages varying in orthographic consistency. *J. Educ. Psychol.* 100 566–580. 10.1037/0022-0663.100.3.566

[B12] Georgiou G. K. Parrila R. Papadopoulos T. C. (2016). The anatomy of the RAN-reading relationship. *Read. Writ.* 29 1793–1815. 10.1016/j.neuroscience.2015.07.071 26235433

[B13] Gong M. Xuan Y. Smart L. J. Olzak L. A. (2018). The extraction of natural scene gist in visual crowding. *Sci. Rep.* 8:14073. 10.1038/s41598-018-32455-6 30232470 PMC6145949

[B14] Gori S. Facoetti A. (2015). How the visual aspects can be crucial in reading acquisition? The intriguing case of crowding and developmental dyslexia. *J. Vis.* 15:15.1.8. 10.1167/15.1.8 25589292

[B15] Guan C. Q. Liu Y. Chan D. H. L. Ye F. Perfetti C. A. (2011). Writing strengthens orthography and alphabetic-coding strengthens phonology in learning to read Chinese. *J. Educ. Psychol.* 103 509–522. 10.1037/a0023730

[B16] Hakvoort B. van den Boer M. Leenaars T. Bos P. Tijms J. (2017). Improvements in reading accuracy as a result of increased interletter spacing are not specific to children with dyslexia. *J. Exp. Child Psychol.* 164 101–116. 10.1016/j.jecp.2017.07.010 28810134

[B17] Hu C. F. (2003). Phonological memory, phonological awareness, and foreign language word learning. *Lang. Learn.* 53 429–462. 10.1111/1467-9922.00231 27175054

[B18] Huang H. S. Hanley J. R. (1995). Phonological awareness and visual skills in learning to read Chinese and English. *Cognition* 54 73–98. 10.1016/0010-0277(94)00641-w 7851080

[B19] Hudson R. F. Lane H. B. Pullen P. C. (2005). Reading fluency assessment and instruction: What, why, and how? *Read. Teach.* 58 702–714. 10.1598/rt.58.8.1

[B20] Hulme C. Hatcher P. J. Nation K. Brown A. Adams J. Stuart G. (2002). Phoneme awareness is a better predictor of early reading skill than onset-rime awareness. *J. Exp. Child Psychol.* 82 2–28. 10.1006/jecp.2002.2670 12081455

[B21] Jones M. W. Branigan H. P. Kelly M. L. (2009). Dyslexic and nondyslexic reading fluency: rapid automatized naming and the importance of continuous lists. *Psychon. Bull. Rev.* 16 567–572. 10.3758/PBR.16.3.567 19451386

[B22] Katzir T. Kim Y. Wolf M. O’Brien B. Kennedy B. Lovett M. (2006). Reading fluency: the whole is more than the parts. *Ann. Dyslexia* 56 51–82. 17849208 10.1007/s11881-006-0003-5

[B23] Kibby M. Y. Lee S. E. Dyer S. M. (2014). Reading performance is predicted by more than phonological processing. *Front. Psychol.* 5:960. 10.3389/fpsyg.2014.00960 25285081 PMC4168686

[B24] Kirby J. R. Roth L. Desrochers A. Lai S. S. V. (2008). Longitudinal predictors of word reading development. *Can. Psychol.* 49 103–110. 10.1037/0708-5591.49.2.103

[B25] Landerl K. Freudenthaler H. H. Heene M. De Jong P. F. Desrochers A. Manolitsis G. (2019). Phonological awareness and rapid automatized naming as longitudinal predictors of reading in five alphabetic orthographies with varying degrees of consistency. *Sci. Stud. Read.* 23 220–234. 10.1080/10888438.2018.1510936

[B26] Leong C. K. Tse S. K. Loh K. Y. Hau K. T. (2008). Text comprehension in Chinese children: relative contribution of verbal working memory, pseudoword reading, rapid automatized naming, and onset-rime phonological segmentation. *J. Educ. Psychol.* 100 135–149. 10.1037/0022-0663.100.1.135

[B27] Levi D. M. Hariharan S. Klein S. A. (2002). Suppressive and facilitatory spatial interactions in peripheral vision: peripheral crowding is neither size invariant nor simple contrast masking. *J. Vis.* 2 167–177. 10.1167/2.2.3 12678590

[B28] Liao C.-H. Georgiou G. K. Parrila R. (2007). Rapid naming speed and Chinese character recognition. *Read. Writ.* 21 231–253.

[B29] Liu Y. Georgiou G. K. Zhang Y. Li H. Liu H. Song S. (2017). Contribution of cognitive and linguistic skills to word-reading accuracy and fluency in Chinese. *Int. J. Educ. Res.* 82 75–90. 10.1016/j.ijer.2016.12.005

[B30] Martelli M. Di Filippo G. Spinelli D. Zoccolotti P. (2009). Crowding, reading, and developmental dyslexia. *J. Vis.* 9 14.1–14.18. 10.1167/9.4.14 19757923

[B31] Mason M. (1975). Reading ability and letter search time: effects of orthographic structure defined by single-letter positional frequency. *J. Exp. Psychol. Gen.* 104 146–166.

[B32] Mather N. Woodcock R. W. (2001). *Woodcock-Johnson III Tests of Cognitive Abilities Examiner’s Manual.* Itasca, IL: Riverside Publishing Company.

[B33] McBride-Chang C. Bialystok E. Chong K. K. Y. Li Y. P. (2004). Levels of phonological awareness in three cultures. *J. Exp. Child Psychol.* 89 93–111. 10.1016/j.jecp.2004.05.001 15388300

[B34] McCallum R. S. Bell S. M. Wood M. S. Below J. L. Choate S. M. McCane S. J. (2006). What is the role of working memory in reading relative to the big three processing variables (orthography, phonology, and rapid naming)? *J. Psychoeduc. Assess.* 24 243–259. 10.1177/0734282906287938

[B35] Meyer M. S. Felton R. H. (1999). Repeated reading to enhance fluency: old approaches and new directions. *Ann. Dyslexia* 49 283–306.

[B36] Morris R. D. Lovett M. W. Wolf M. Sevcik R. A. Steinbach K. A. Frijters J. C. (2012). Multiple-component remediation for developmental reading disabilities: IQ, socioeconomic status, and race as factors in remedial outcome. *J. Learn. Disabil.* 45 99–127. 10.1177/0022219409355472 20445204 PMC9872281

[B37] Nathan R. G. Stanovich K. E. (1991). The causes and consequences of differences in reading fluency. *Theory Pract.* 30 176–184. 10402967

[B38] Norton E. S. Wolf M. (2012). Rapid automatized naming (RAN) and reading fluency: implications for understanding and treatment of reading disabilities. *Annu. Rev. Psychol.* 63 427–452. 10.1146/annurev-psych-120710-100431 21838545

[B39] O’Brien B. A. Wolf M. Miller L. T. Lovett M. W. Morris R. (2011). Orthographic processing efficiency in developmental dyslexia: an investigation of age and treatment factors at the sublexical level. *Ann. Dyslexia* 61 111–135. 10.1007/s11881-010-0050-9 21213077 PMC11537046

[B40] Oldfield R. C. (1971). The assessment and analysis of handedness: the edinburgh inventory. *Neuropsychologia* 9 97–113. 5146491 10.1016/0028-3932(71)90067-4

[B41] Palmer M. L. (2010). The Relationship between Reading Fluency, Writing Fluency, and Reading Comprehension in Suburban Third-Grade Students. Third-Grade Students, ProQuest Digital dissertation, University of San Diego, San Diego, CA.

[B42] Papadopoulos T. C. Spanoudis G. C. Georgiou G. K. (2016). How is RAN related to reading fluency? A comprehensive examination of the prominent theoretical accounts. *Front. Psychol.* 7:1217. 10.3389/fpsyg.2016.01217 27605918 PMC4995210

[B43] Pasquarella A. Chen X. Gottardo A. Geva E. (2015). Cross-language transfer of word reading accuracy and word reading fluency in Spanish-English and Chinese-English bilinguals: script-universal and script-specific processes. *J. Educ. Psychol.* 107 96–110. 10.1037/a0036966

[B44] Pelli D. G. (2008). Crowding: a cortical constraint on object recognition. *Curr. Opin. Neurobiol.* 18 445–451. 10.1016/j.conb.2008.09.008 18835355 PMC3624758

[B45] Pelli D. G. Palomares M. Majaj N. J. (2004). Crowding is unlike ordinary masking: distinguishing feature integration from detection. *J. Vis.* 4 1136–1169. 10.1167/4.12.12 15669917

[B46] Pelli D. G. Tillman K. A. Freeman J. Su M. Berger T. D. Majaj N. J. (2007). Crowding and eccentricity determine reading rate. *J. Vis.* 7 20.1–36. 10.1167/7.2.20 18217835

[B47] Perfetti C. (1977). “Language comprehension and fast decoding: some psycholinguistic prerequisites for skilled reading comprehension,” in *Cognition, Curriculum and Comprehension*, ed. Guthrie J. T. (Newark, DE: International Reading Association).

[B48] Perfetti C. (1985). *Reading Ability.* Oxford: Oxford University Press.

[B49] Perfetti C. Cao F. Booth J. (2013). Specialization and universals in the development of reading skill: how Chinese research informs a universal science of reading. *Sci. Stud. Read.* 17 5–21. 10.1080/10888438.2012.689786 24744605 PMC3987914

[B50] Poulsen M. Elbro C. (2013). What’s in a name depends on the type of name: the relationships between semantic and phonological access, reading fluency, and reading comprehension. *Sci. Stud. Read.* 17 303–314. 10.1080/10888438.2012.692743

[B51] Protopapas A. Altani A. Georgiou G. K. (2013). Development of serial processing in reading and rapid naming. *J. Exp. Child Psychol.* 116 914–929. 10.1016/j.jecp.2013.08.004 24077466

[B52] Rakhlin N. Cardoso-Martins C. Grigorenko E. L. (2014). Phonemic awareness is a more important predictor of orthographic processing than rapid serial naming: evidence from Russian. *Sci. Stud. Read.* 18 395–414. 10.1080/10888438.2014.918981 25435759 PMC4243176

[B53] Rakhlin N. V. Mourgues C. Cardoso-Martins C. Kornev A. N. Grigorenko E. L. (2019). Orthographic processing is a key predictor of reading fluency in good and poor readers in a transparent orthography. *Contemp. Educ. Psychol.* 56 250–261. 10.1016/j.cedpsych.2018.12.002 31798206 PMC6890420

[B54] Savage R. Frederickson N. (2005). Evidence of a highly specific relationship between rapid automatic naming of digits and text-reading speed. *Brain Lang.* 93 152–159. 10.1016/j.bandl.2004.09.005 15781303

[B55] Schatschneider C. Fletcher J. M. Francis D. J. Carlson C. D. Foorman B. R. (2004). Kindergarten prediction of reading skills: a longitudinal comparative analysis. *J. Educ. Psychol.* 96 265–282. 10.1037/0022-0663.96.2.265

[B56] Shankweiler D. Crain S. (1986). Language mechanisms and reading disorder: a modular approach. *Cognition* 24 139–168. 3791919 10.1016/0010-0277(86)90008-9

[B57] Siok W. T. Fletcher P. (2001). The role of phonological awareness and visual-orthographic skills in Chinese reading acquisition. *Dev. Psychol.* 37 886–899. 10.1037//0012-1649.37.6.886 11699761

[B58] Siok W. T. Perfetti C. A. Jin Z. Tan L. H. (2004). Biological abnormality of impaired reading is constrained by culture. *Nature* 431 71–76. 10.1038/nature02865 15343334

[B59] Sodoro J. Allinder R. M. Rankin-Erickson J. L. (2002). Assessment of phonological awareness: review of methods and tools. *Educ. Psychol. Rev.* 14 223–260. 10.1023/a:1016050412323

[B60] Song S. Georgiou G. K. Su M. Hua S. (2016). How well do phonological awareness and rapid automatized naming correlate with chinese reading accuracy and fluency? A meta-analysis. *Sci. Stud. Read.* 20 99–123. 10.1080/10888438.2015.1088543

[B61] Strappini F. Pelli D. G. Di Pace E. Martelli M. (2017). Agnosic vision is like peripheral vision, which is limited by crowding. *Cortex* 89 135–155. 10.1016/j.cortex.2017.01.012 28284488 PMC8493918

[B62] Tan L. H. Spinks J. A. Eden G. F. Perfetti C. A. Siok W. T. (2005). Reading depends on writing, in Chinese. *Proc. Natl. Acad. Sci. U.S.A.* 102 8781–8785. 10.1073/pnas.0503523102 15939871 PMC1150863

[B63] Tan L. H. Xu M. Chang C. Q. Siok W. T. (2013). China’s language input system in the digital age affects children’s reading development. *Proc. Natl. Acad. Sci. U.S.A.* 110 1119–1123. 23277555 10.1073/pnas.1213586110 PMC3549123

[B64] Toet A. Levi D. M. (1992). The 2-dimensional shape of spatial interaction zones in the parafovea. *Vision Res.* 32 1349–1357. 10.1016/0042-6989(92)90227-a 1455707

[B65] Tso R. V. Y. Au T. K. Hsiao J. H. (2012). Writing facilitates learning to read in Chinese through reduction of holistic processing: a developmental study. *J. Vis.* 12 530–530.

[B66] Vaessen A. Bertrand D. Tóth D. Csépe V. Faísca L. Reis A. (2010). Cognitive development of fluent word reading does not qualitatively differ between transparent and opaque orthographies. *J. Educ. Psychol.* 102 827–842. 10.1037/a0019465

[B67] van den Bos K. P. (1998). IQ, phonological awareness and continuous-naming speed related to Dutch poor decoding children’s performance on two word identification tests. *Dyslexia* 4 73–89.

[B68] Van Yip Tso R. A. Hsiao T. K. F. Hui-we J. (2012). Writing facilitates learning to read in Chinese through reduction of holistic processing: a developmental study. *Proc. Annu. Meet. Cogn. Sci. Soc.* 34:530 10.1167/12.9.530

[B69] Wagner R. K. Torgesen J. K. (1987). The nature of phonological processing and its causeal role in the acquisition of reading-skills. *Psychol. Bull.* 101 192–212. 10.1037/0033-2909.101.2.192

[B70] Wang H. He X. Legge G. E. (2014). Effect of pattern complexity on the visual span for Chinese and alphabet characters. *J. Vis.* 14:6. 10.1167/14.8.6 24993020 PMC4083876

[B71] Whitney D. Levi D. M. (2011). Visual crowding: a fundamental limit on conscious perception and object recognition. *Trends Cogn. Sci.* 15 160–168. 10.1016/j.tics.2011.02.005 21420894 PMC3070834

[B72] Wolf M. Bally H. Morris R. (1986). Automaticity, retrieval-processes, and reading-a longitudinal-study in average and impaired readers. *Child Dev.* 57 988–1000. 10.1111/j.1467-8624.1986.tb00260.x 3757613

[B73] Wolf M. Barzillai M. Gottwald S. Miller L. Spencer K. Norton E. (2009). The RAVE-O intervention: connecting neuroscience to the classroom. *Mind Brain Educ.* 3 84–93. 10.1111/j.1751-228X.2009.01058.x PMC11456325 39372597

[B74] Wolf M. Bowers P. G. (1999). The double-deficit hypothesis for the developmental dyslexias. *J. Educ. Psychol.* 91 415–438. 10.1037/0022-0663.91.3.415

[B75] Xue J. Shu H. Li H. Li W. Tian X. (2013). The stability of literacy-related cognitive contributions to chinese character naming and reading fluency. *J. Psycholinguist. Res.* 42 433–450. 10.1007/s10936-012-9228-0 22923217

[B76] Zhang J.-Y. Zhang T. Xue F. Liu L. Yu C. (2009). Legibility of Chinese characters in peripheral vision and the top-down influences on crowding. *Vision Res.* 49 44–53. 10.1016/j.visres.2008.09.021 18929592

[B77] Ziegler J. C. Bertrand D. Toth D. Csepe V. Reis A. Faisca L. (2010). Orthographic depth and its impact on universal predictors of reading: a cross-language investigation. *Psychol. Sci.* 21 551–559. 10.1177/0956797610363406 20424101

[B78] Ziegler J. C. Goswami U. (2005). Reading acquisition, developmental dyslexia, and skilled reading across languages: a psycholinguistic grain size theory. *Psychol. Bull.* 131 3–29. 10.1037/0033-2909.131.1.3 15631549

[B79] Zorzi M. Barbiero C. Facoetti A. Lonciari I. Carrozzi M. Montico M. (2012). Extra-large letter spacing improves reading in dyslexia. *Proc. Natl. Acad. Sci. U.S.A.* 109 11455–11459. 10.1073/pnas.1205566109 22665803 PMC3396504

